# Stuttering Thoughts: Negative Self-Referent Thinking Is Less Sensitive to Aversive Outcomes in People with Higher Levels of Depressive Symptoms

**DOI:** 10.3389/fpsyg.2017.01333

**Published:** 2017-08-02

**Authors:** Yudai Iijima, Keisuke Takano, Yannick Boddez, Filip Raes, Yoshihiko Tanno

**Affiliations:** ^1^Graduate School of Education, University of Tokyo Tokyo, Japan; ^2^Center for Learning and Experimental Psychopathology, University of Leuven Leuven, Belgium; ^3^Graduate School of Arts and Sciences, University of Tokyo Tokyo, Japan

**Keywords:** self-reference, depression, reinforcement learning, Q-learning model, rumination

## Abstract

Learning theories of depression have proposed that depressive cognitions, such as negative thoughts with reference to oneself, can develop through a reinforcement learning mechanism. This negative self-reference is considered to be positively reinforced by rewarding experiences such as genuine support from others after negative self-disclosure, and negatively reinforced by avoidance of potential aversive situations. The learning account additionally predicts that negative self-reference would be maintained by an inability to adjust one’s behavior when negative self-reference no longer leads to such reward. To test this prediction, we designed an adapted version of the reversal-learning task. In this task, participants were reinforced to choose and engage in either negative or positive self-reference by probabilistic economic reward and punishment. Although participants were initially trained to choose negative self-reference, the stimulus-reward contingencies were reversed to prompt a shift toward positive self-reference (Study 1) and a further shift toward negative self-reference (Study 2). Model-based computational analyses showed that depressive symptoms were associated with a low learning rate of negative self-reference, indicating a high level of reward expectancy for negative self-reference even after the contingency reversal. Furthermore, the difficulty in updating outcome predictions of negative self-reference was significantly associated with the extent to which one possesses negative self-images. These results suggest that difficulty in adjusting action-outcome estimates for negative self-reference increases the chance to be faced with negative aspects of self, which may result in depressive symptoms.

## Introduction

Four decades of studies have shown that individuals with clinical and subclinical depressive symptoms have a negativity bias in self-referent information processing. Cognitive models of depression have highlighted the negative views of the self, the world, and the future as the cognitive triad of depression (e.g., Beck, 1976; Ingram, 1990). Dysfunctions in self-referent information processing have gained particular attention in social and clinical psychology, as early studies demonstrated that an excessive degree of self-focused attention is correlated with increased levels of depressive symptoms (e.g., Smith and Greenberg, 1981; Ingram and Smith, 1984). More recent studies have confirmed that negative automatic thoughts and biases in attention, interpretation, and memory are associated with depression (Mathews and MacLeod, 2005; Gotlib and Joormann, 2010; LeMoult et al., 2016), particularly when the stimuli are self-relevant or processed in a self-relevant manner (e.g., Mogg and Bradley, 2005; Joormann and Tran, 2009).

Other studies have suggested that individuals with depressive symptoms tend to lack a positivity bias that non-depressed individuals have; although people generally tend to attribute positive (rather than negative) matters to internal, stable, and global factors, this tendency is weak or absent in people with depressive symptoms (Mezulis et al., 2004). The positivity bias could function protectively to divert attention away from negative information and to direct it to positive information; therefore shielding people from negative self-referent processing and preserving positive self-views (Gotlib et al., 1988; McCabe and Gotlib, 1995; McCabe et al., 2000). Excessive focus on negative aspects of the self (i.e., negative self-referent processing) and lack of focus on its positive aspects is associated with unbalanced accessibility of negative and positive self-referent materials, thereby contributing to depressive rumination (e.g., Trew, 2011).

Despite a large number of studies that have investigated the altered emotional self-referent processing in depression, the mechanisms underlying the self-negativity biases and the absence of self-positivity biases are still subjects of ongoing debate. How should we understand these biases? One important aspect, and the focus of the current study, is the inflexibility in adjusting cognition and behavior to a changing environment. Previous studies have suggested that individuals with depressive symptoms tend to have difficulty refreshing working memory by eliminating information that is no longer relevant (Joormann and Gotlib, 2008; Levens and Gotlib, 2010; Pe et al., 2016), inhibiting negative information processing (Joormann, 2004; Goeleven et al., 2006), and disengaging current attention from negative stimuli (Koster et al., 2005; Mogg and Bradley, 2005; Leyman et al., 2007). Such cognitive inflexibility in updating attention and memory explains why people with depressive symptoms experience difficulty in stopping their negative self-referent thinking.

The updating function has also been examined from a learning perspective, suggesting that the inability to update a current belief on action-outcome contingencies is associated with heightened levels of depressive and anxiety symptoms. If individuals with high levels of depressive symptoms are provided with inaccurate instructions about how to succeed at a learning task, they showed persistent and problematic rule-following behaviors throughout the task (McAuliffe et al., 2014). Similarly, trait anxiety is associated with the inability to update action-outcome estimates following unexpected aversive outcomes (Browning et al., 2015). These findings suggest that individuals with depression (and anxiety) symptoms have difficulty in adjusting their beliefs in a volatile environment (where the outcomes are not static but are changeable) once these beliefs have been learned and established. Such inflexibility in updating beliefs or action-outcome predictions could well explain the development and maintenance processes of self-negativity bias in depression, since it is possible that (a) negative self-referent thinking is learned and reinforced, and that (b) negative self-reference is persistently perceived to be accompanied with reward even after environmental changes.

Indeed, the learning theory of depression suggests that the development of depressive cognitions can be explained by reinforcement learning principles (Watkins and Nolen-Hoeksema, 2014; Ramnerö et al., 2015). More precisely, this account holds that the repeated presentation of reward after a negative thought will increase the frequency of such negative thoughts. Although negative self-referent thinking has clear adverse consequences, such as increasing negative affect, negative self-reference can be perceived to have beneficial outcomes that function as reward in specific contexts. For example, complaining and expressing negative aspects of the self may be initially reinforced by the genuine support and concern of other people (e.g., Ramnerö et al., 2015), even if excessive disclosure about negative aspects of the self might cause fatigue and social rejection by others in a long-term relationship (Coyne, 1976). Self-focused thinking may also be perceived as a means to enhance self-knowledge and to generate possible solutions in difficult situations. In line with this, self-reflection may indeed help understand oneself and analyze problems accurately (e.g., Trapnell and Campbell, 1999; Watkins, 2008).

In addition to positive reinforcement, negative reinforcement might also play an important role in the development of self-negativity bias in depression. It has been argued that depressive rumination (a repetitive and persistent form of negative self-focused thinking) functions as an act of avoidance, which can temporally reduce emotional distresses by preventing even more aversive situations and the responsibility to take action (Nolen-Hoeksema et al., 2008; Watkins and Nolen-Hoeksema, 2014). However, such avoidance also prevents actual problem-solving and the opportunity to experience that certain situations may actually be not so aversive or even rewarding, thereby sometimes contributing to the depressed state in the long-term (Jacobson et al., 2001; Ramnerö et al., 2015).

Models of reinforcement learning propose that the efficiency of the updating function can be represented by a parameter that quantifies the learning rate. In the reinforcement-learning framework, an agent (a) predicts reward values of action candidates (e.g., pressing a right vs. a left key), (b) selects an action that maximizes the predicted reward value (e.g., amount of juice or money), and (c) updates the predictions according to the consequences of the action (e.g., Daw and Doya, 2006; Corrado and Doya, 2007; O’Doherty et al., 2007). Learning progresses through minimizing the mismatch between the actual and predicted outcomes of an action (i.e., prediction error) and maximizing the predicted value that is associated with the current action. In other words, the current action-outcome estimate is determined by the previous prediction error, which is weighted by a learning rate that varies depending on individual and environmental factors. The learning rate becomes smaller when an agent is less sensitive to environmental changes, that is, when the agent’s action-outcome estimates are more slowly updated. In the present study, we hypothesized that individuals with depressive symptoms would have a low learning rate for negative self-reference. A low learning rate represents difficulty in discarding the learned beliefs that negative self-reference brings beneficial outcomes *even when* the actual outcomes are harmful. Such inflexible adjustment in action-outcome predictions would contribute to the excessive use of negative self-reference.

Therefore, we designed a laboratory model that allowed studying the development and maintenance of the self-negativity bias. More specifically, we examined the relationship between individual differences in depressive symptoms and the learning of associations between emotional self-reference and related reward by using an adapted version of the probabilistic reversal learning (RL) task. In a typical RL task, participants are offered two options, one associated with higher probability of reward and the other associated with higher probability of punishment. Over the course of training, participants learn which response instrumentally generates a reward (Izquierdo and Jentsch, 2012). However, in the middle of the task, the stimulus-reward contingencies are reversed. As the trained response no longer results in a reward, participants have to discount the outcome prediction of the option that was initially a “correct” response and have to switch to the other response.

**FIGURE 1:**
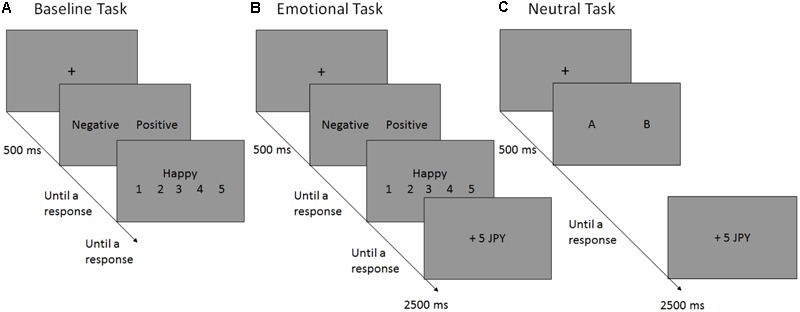
Schematic flow of a single trial in the baseline **(A)**, emotional **(B)** and neutral **(C)** reversal learning tasks. In each experiment, the participants performed the baseline task **(A)**, in which they selected the preferred valence (“negative” vs. “positive”) for the following self-referent rating [e.g., Questions such as “Happy?” (or “Unhappy?”) were displayed if the “positive” (or “negative”) option were selected]. In the emotional reversal learning task **(B)**, participants selected the preferred valence for the following self-referent rating and, subsequently, monetary reward (+5 JPY) or punishment (–5 JPY) was presented probabilistically, depending on the participants’ valence choices. The reinforcement schedules have been presented in **Figure 2**. In the neutral reversal learning task **(C)**, the participants choose between the letters “A” and “B”, which was rewarded or punished according to the same reinforcement schedule used in the emotional RL task. JPY, Japanese yen.

In our adapted version of the RL task, participants were offered two emotional (negative vs. positive) options. Depending on their choice, participants were expected to engage in negative or positive self-reference (**Figure 1**). More precisely, participants were presented with a self-attribute that had the same valence as their choice, and were asked to rate to what extent the self-attribute was applicable to them (Tamir and Mitchell, 2012; Takano et al., 2016). For example, if a participant chose the “positive” option, they had to rate the applicability of a positive attribute (e.g., “Happy”). Immediately after the rating, either a reward or punishment was presented probabilistically, depending on the participant’s valence choice. In the acquisition phase, participants learned that the “negative” option was more likely to generate a reward than the “positive” counterpart; however, in the reversal phase, the “negative” option was no longer the correct response, and was more likely to be followed by punishment. To perform this task well, participants had to update the outcome prediction of negative self-reference efficiently after the contingency reversal. As described above, we predicted that depressive symptoms would be associated with a significant delay in updating the outcome prediction of negative self-reference.

This hypothesis was tested in two studies using the emotional RL task. In Study 1, participants were initially trained to choose negative self-reference (acquisition phase), following which, they were to choose positive self-reference to obtain reward (reversal phase). This task allowed us to examine the process of discounting the reward prediction of negative self-reference in the reversal phase. However, because Study 1 did not cover the transition from positive to negative self-reference, we could not examine the re-learning process of negative-reward associations (i.e., increasing the reward prediction of negative self-reference). This was particularly important for individuals with higher levels of depressive symptoms, who were expected to have greater preference toward negative self-reference already during initial trials of the acquisition phase. Therefore, in Study 2, we added the second reversal phase, in which participants were trained to choose negative self-reference again after being trained to choose positive self-reference.

As an additional hypothesis, we tested the mediational role of self-image in the association between the updating of reward prediction and the level of depressive symptoms. We predicted that difficulty in updating reward prediction for negative self-reference would be associated with more negative self-images (e.g., because persistently collecting and therefore being exposed to negative self-referent information leads to negative self-views and/or decreases positive self-views) and that the reinforced negative self-views may contribute to depressive symptoms. More precisely, we hypothesized that the association between the low learning rate for negative self-reference and depressive symptoms would be mediated by a more negative (and less positive) self-image (i.e., by a higher applicability rating score for negative attributes and a lower score for positive attributes during the emotional RL task).

## Study 1

### Method

#### Participants

Thirty-nine participants (16 men and 23 women; mean age = 19.6 years, *SD* = 2.8 years) were recruited from a large sample pool of undergraduate students from the University of Tokyo. No specific inclusion/exclusion criteria were used.

#### Measure

Participants completed the Japanese version of the Center for Epidemiologic Studies Depression Scale (CES-D; Radloff, 1977; Shima et al., 1985), which is a 20-item self-report questionnaire that measures the levels of depressive symptoms during the previous week. Each item describes a typical symptom of depression and is rated on a four-point scale of the frequency of occurrence ranging from 0 (*less than 1 day*) to 3 (*5–7 days*). The mean CES-D score was 11.9 (*SD* = 7.9) and the Cronbach’s α was 0.88. Ten participants had clinically significant levels of symptoms, which exceeded the cut-off score of the CES-D (>15; Radloff, 1977).

#### The Baseline Task

Before performing the emotional and neutral RL tasks, participants completed a baseline task (**Figure 1A**) to assess the preference for negative self-reference that participants originally had. In this task, participants were presented with a positive and a negative valence option. They were asked to choose either of the two options, following which an attribute corresponding to the selected valence was displayed (if the “positive” option was selected, an attribute, such as “Happy,” was presented). Participants were instructed to rate the extent to which the presented attribute was applicable to them on a five-point scale ranging from (1) *not at all* to (5) *very much*. Therefore, if a participant preferred negative self-reference, he/she would choose the “negative” option more frequently than the “positive” option. When making the preference choices, participants were informed only of the valence types (i.e., “Negative” vs. “Positive”), but the specific content of the attributes (e.g., “Unhappy” vs. “Happy”) was blinded until the attribute-rating display appeared. Participants completed 20 trials in the baseline task.

We used a list of negative and positive attributes, a subset of which was used in a previous study (Takano et al., 2016). The list comprised 100 pairs of negative and positive attributes that produce bipolar sets of traits (e.g., happy vs. unhappy, arrogant vs. humble, frequently having troubles with family members vs. having a good relationship with family members)^1^. The length of stimuli between the negative and positive counterparts was matched, but it was not controlled within negative (or positive) materials. This is because (a) we adopted the items from existing questionnaires of personality, depression, anxiety, and social functioning (see Takano et al., 2016), and (b) there should be no or little influence of stimulus length given that we did not impose a strict response time window. In each trial, one of the attributes was randomly selected. Among the 100 pairs, 20 were used in the baseline task, and the other 80 pairs were used in the following emotional RL task. All attributes had been confirmed to have negative or positive valence by two psychological researchers who were unaware of the aim of the present study.

#### The Emotional Reversal Learning (RL) Task

**FIGURE 2:**
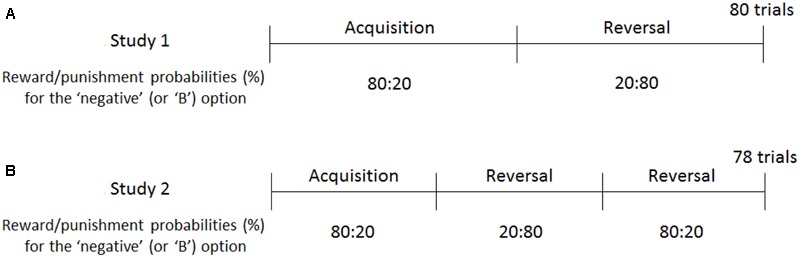
Reward and punishment schedules of Experiments 1 and 2. In Study 1 **(A)**, participants were trained to choose negative self-reference in the first half of the trials, following which they were prompted to shift toward positive self-reference in the reversal phase. In Study 2 **(B)**, the contingency reversal took place twice to train the participants to select negative, positive, and negative self-reference. The reward/punishment probabilities were opposite for the “positive” and “A” options (e.g., 20:80 in the acquisition phase).

Similar to the baseline task, participants were asked to choose either the negative or positive valence option in each trial. Depending on the participants’ valence choice, a positive or negative attribute was displayed. Participants rated to what extent the displayed attribute was applicable to them using a five-point Likert scale as in the baseline task. After the rating, feedback of reward (+5 JPY) or punishment (-5 JPY) was displayed probabilistically, depending on the valence choice (1 JPY = 0.01 USD). The task consisted of 80 trials; the first 40 trials were the acquisition phase, in which the “negative” option was more associated with reward than with punishment (at a 80:20% probability); the latter half was the reversal phase, in which the “negative” option was more associated with punishment than with reward (at a 20:80% probability; **Figure 2A**). The “positive” option had the opposite reinforcement schedule; the probabilities of reward and punishment were 20:80% in the acquisition and 80:20% in the reversal phase. Before starting this task, participants were informed that: (a) they would be paid the total amount of money acquired during the task; (b) the reward and punishment were determined by the valence choice but not by the attribute rating^2^; (c) either the negative or positive valence option is more likely to be associated with reward than punishment (and vice versa); and (d) the contingency would be changed during the experiment, although the timing and the number of the changes were not mentioned. Participants were not explicitly instructed to maximize the total amount of reward.

#### The Neutral Reversal Learning (RL) Task

To assess and control the general learning (and value updating) ability, we administered the neutral RT task with non-emotional and non-self-referent stimuli. Participants chose between the letters A and B, which was probabilistically rewarded and punished as per the same reinforcement schedule that was used in the emotional RL task. This neutral task also consisted of 80 trials; the first half was the acquisition phase, in which the “B” option was more associated with reward than was the “A” option; the latter half was the reversal phase, in which the “A” option was more associated with reward than was the “B” option. Participants were informed that (a) they would be paid the total amount of money that they acquired in this task and in the emotional RL task, and that (b) the reward-punishment feedback is probabilistically determined by the A–B choice. As in the emotional RL task, they were not explicitly instructed to maximize the total amount of reward.

#### Procedure

Participants were invited to the laboratory individually. On arrival, they provided written informed consent. First, participants completed the baseline task, following which they completed the emotional and neutral RL tasks. The order of the emotional and neutral RL tasks was counterbalanced across participants. Finally, the participants completed a self-report questionnaire to measure depressive symptoms, and were debriefed and paid the amount of money acquired during the RL tasks. All study protocols were approved by the Ethical Committee for Experimental Research on Human Subjects of the University of Tokyo.

#### Statistical Analyses

We employed the Q-learning model (Watkins and Dayan, 1992; Sutton and Barto, 1998) to extract specific features (i.e., learning rate) of the participants’ individual learning processes in the reversal learning tasks. The Q-learning model assumes that the participants’ choice behavior is determined by outcome predictions of choosing either of the two options (i.e., negative vs. positive self-references, in the emotional RL task; B and A in the neutral task). The outcome predictions are updated in each trial by the difference between the actual outcome (reward or punishment) and expected value of the chosen option, namely the prediction error of the Rescorla-Wagner rule. In our model of the emotional RL task, the updating processes of the outcome predictions were represented as follows:

For trials in which negative self-reference was chosen:

$$Q_{neg}(t+1)=Q_{neg}(t)+\alpha_{neg}(R(t)-Q_{neg}(t))$$

For trials in which positive self-reference was chosen:

$$Q_{pos}(t+1)=Q_{pos}(t)+\alpha_{pos}(R(t)-Q_{pos}(t))$$

where *Q_neg_* (*t* + 1) and *Q_pos_* (*t* + 1) are the outcome predictions of the two choice options (negative and positive self-reference) at trial *t* + 1. These outcome predictions are determined by the prediction error represented by the difference between the actual reward, *R*(*t*), and the outcome prediction, *Q_neg_*(*t*) or *Q_pos_*(*t*) at the previous trial, *t*. Unlike the original Q-learning model, we assumed two learning rates (i.e., *α_neg_* and *α_pos_*) that may be different between the “negative” and “positive” options (double update model; cf. Schlagenhauf et al., 2014). Previous studies have proposed variants of the Q-learning model depending on the tasks and stimuli; for example, assuming different learning rates between rewarded and punished trials (e.g., Dombrovski et al., 2010) and between chosen and unchosen options (e.g., Li and Daw, 2011; Schlagenhauf et al., 2014), and temporally variable learning rates over trials (Bai et al., 2014). The current assumption of the differential learning rates was motivated by the hypothesis that individuals with depressive symptoms would have difficulty in updating the outcome predictions, particularly for negative self-reference. A low learning rate of the “negative” option reflects a slow-down in updating the outcome prediction for negative self-reference because the prediction error at the previous trial has only a small influence on the current outcome prediction. Conversely, a high learning rate indicates that the outcome prediction changes easily in response to prediction error, which results in a quick switch between the two options after the contingency reversal. Importantly, under this assumption, outcome predictions were updated independently for negative and positive self-reference. Since each updating process is solely coded by the corresponding (either negative or positive) learning rate (and exploitation parameter), the learning trajectory of negative and positive self-reference can be described separately. Initial values of the Q parameters were determined by the proportions of “negative” and “positive” choices in the baseline task, which reflect the preference for negative or positive self-reference that each participant originally had.

The probability of choosing the “negative” option at trial *t* is then represented by a sigmoid function of the difference in the outcome predictions between the negative and positive options:

$$P_{neg}(t)=\frac{1}{1-\exp(-\beta (Q_{neg}(t)-Q_{pos}(t)))}$$

where *β* is an exploration-exploitation parameter, which reflects the reinforcement history, with a larger value indicating greater sensitivity to the Q difference between the two options.

This Q-learning model was fitted to the observed choice behaviors of each participant individually. The optimal values of *α_neg_, α_pos_*, and *β* were searched by the log maximum likelihood estimation, in which we calculated the log-sum of the probabilities that the model would select the option that the participant actually selected at trial *t* [*P*(choice *t*)]. Thus, the log likelihood [log(*L*)] was presented as follows:

$$\log(L)=\sum_{t}\log(P(choice_{t}))$$

The log-likelihood was maximized by the Broyden–Fletcher–Goldfarb–Shanno algorithm of the R optimal function under the “hard constraints” on the lower and upper limits of the parameter values (0 ≤*α_neg_, α_pos_* ≤ 1; e.g., Daw, 2011).

We fitted the same three-parameter Q-learning model to the choice behaviors observed in the neutral RL task. Although we did not expect any differences in the learning rates between the option B and A (*α_B_* and *α_A_*, corresponding to *α_neg_* and *α_pos_*), these parameters were separately estimated in order to compare the results of the neutral RL task with those of the emotional RL task. The initial values of the Q parameters were set to be zero, because there would be no clear pre-existing preferences to the neutral stimuli. It is to be noted that the learning parameters (i.e., learning rates and exploitation parameter) were not influenced by the order of the emotional and neutral RL tasks, *t*s < 1.39, *p*s > 0.17.

#### Model Comparison

The goodness of fit of the models was tested by using the Akaike’s information criterion (AIC), presented as follows:

AIC=2log(L)+2k,

where *k* represents the number of free parameters. A smaller AIC value indicates a better model fit. The AIC prefers a parsimonious model because it includes a penalty term that increases as a function of the number of estimated parameters. We compared the AIC of the double update model (i.e., assuming *α_neg_* and *α_pos_*) to that of the single update model (i.e., assuming equal constraints on *α_neg_* and *α_pos_*) in order to verify that the double learning rates explain participants’ choice behaviors better than the single learning rate. However, it is not necessary that all participants have a smaller AIC for the double than the single update model. We expected that there would be individual differences in the balance of the learning rates of the negative and positive self-reference; some individuals would have equal levels of the learning rates between the “negative” and “positive” options, whereas other individuals would have unbalanced learning rates (e.g., reduced learning rate specifically of the “negative” option). **Table 1** shows the results of the model comparison. For approximately one-third of the participants, the double update model explained the data better than did the single update model, in terms of the AIC; however, for the other participants, the single learning rate was sufficient to explain their choice behaviors. Because the single update model is a lower model that is nested in the double update model, we performed the subsequent analyses based on the estimates of the double update model; that is, if depressive symptoms are associated with an impairment in a general updating ability (not specific for negative or positive self-reference), both the learning rates for negative and positive response options should be correlated with depressive symptoms.

**Table 1:** Mean negative log likelihood and AIC across participants for the single and double update models.

	Negative log likelihood		AIC		N of participants with AIC as Double < Single	
	Double	Single	Double	Single	(N/total sample size)
Study 1	21.14	22.00	48.28	48.00	12/39
Study 2	23.02	24.00	52.03	51.98	13/44

*AIC, Akaike’s information criterion.*

#### Sample Size Calculation

We determined sample sizes by power analysis (G^∗^power; Faul et al., 2009). Our main analyses focused on multiple regressions predicting depressive symptoms by the six learning parameters from the emotional *(α_neg_, α_pos_*, and *β*) and neutral RL tasks (*α_B_, α_A_*, and *β*). According to the power analysis, a sample size of *n* = 26–55 is needed to detect a medium-to-large effect of a single regression coefficient (*f^2^* = 0.15–0.35) under the assumption of alpha = 0.05 and beta = 0.80. One previous study examined reinforcement learning in non-clinical depressed samples, showing a medium-to-large effect size for the difference in a learning parameter between high and low depression groups (Hedge’s *g* = 0.73; Kunisato et al., 2012). Based on this result, we set the sample sizes to be approximately 40–50, which enabled us to detect a medium-to-large effect.

### Results

In the baseline task, wherein no feedback of reward and punishment was provided, individuals with higher levels of depressive symptoms were more likely to choose negative self-reference (*r* = 0.31, *p* = 0.05). Those with higher levels of depressive symptoms rated the negative attributes to be more applicable (*r* = 0.49, *p* < 0.01) and positive attributes to be less applicable to them (*r* = -0.50, *p* < 0.01)^3^. These tendencies were also observed in the emotional RL task (*r* = 0.69, *p* < 0.01 for negative self-reference; *r* = -0.38, *p* = 0.02 for positive self-reference).

In the emotional RL task, all participants performed better than chance in the acquisition and reversal phases (**Table 2**). These results suggest that participants were successful at learning the initial association between negative self-reference and reward, and they could subsequently adapt their behavior to choose positive self-reference in accordance with the contingency reversal. As a possible strategy, participants could always rate negative attributes being “not at all” applicable to themselves in order to avoid negative self-reference (and could rate positive attributes as not being applicable to avoid positive self-reference). Therefore, we examined the frequency of the “*not at all*” response, which was only 10.3% across all trials in the task. Thus, participants retrieved the aspects of the self that corresponded to the displayed self-attributes in most (90%) of the trials.

**Table 2:** Means and SDs of choice frequencies of negative self-reference and learning parameters in the baseline, emotional, and neutral reversal learning (RL) tasks for Studies 1 and 2.

	Study 1 (*n* = 39)		Study 2 (*n* = 44)	
	*M*	*SD*	*M*	*SD*
Baseline task—choice frequency	0.40	0.23	0.42	0.24
Emotional RL task				
Choice frequency				
Acquisition	0.81	0.11	0.78	0.15
First reversal	0.21	0.19	0.23	0.12
Second reversal	–	–	0.78	0.11
Learning rate—negative (*α_neg_*)	0.60	0.28	0.70	0.26
Learning rate—positive (*α_pos_*)	0.58	0.30	0.71	0.24
Exploitation (*β*)	12.06	11.26	8.33	8.20
Neutral RL task				
Choice frequency				
Acquisition	0.77	0.14	0.79	0.16
First reversal	0.21	0.14	0.23	0.15
Second reversal	–	–	0.79	0.14
Learning rate—B (*α_B_*)	0.69	0.28	0.74	0.23
Learning rate—A (*α_A_*)	0.52	0.30	0.66	0.30
Exploitation (*β*)	11.60	11.75	15.53	13.09

**FIGURE 3:**
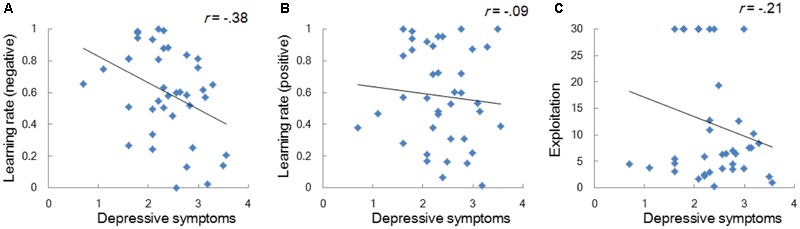
Correlations between depressive symptoms and the learning rates of negative **(A)** and positive self-reference **(B)**, and the exploitation parameter **(C)** in Study 1. The depressive symptoms score (the CES-D score) was log transformed.

**FIGURE 4:**
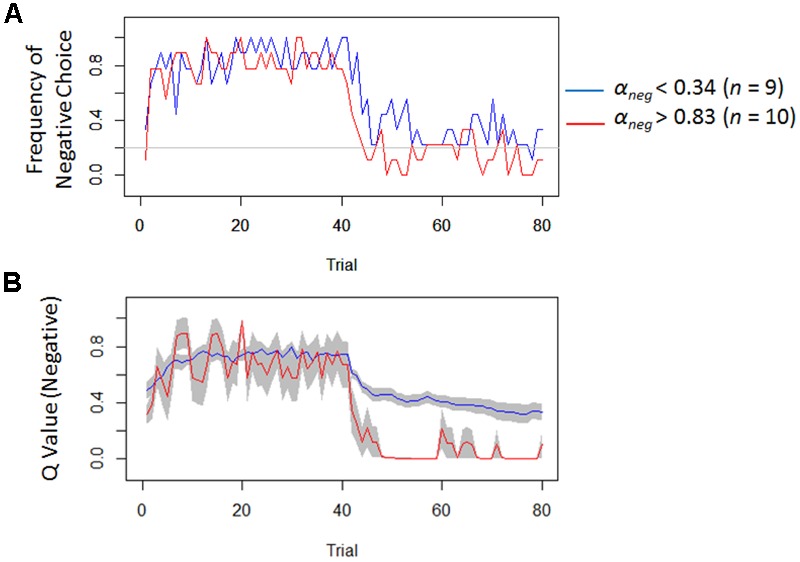
Frequency of negative choice and average outcome predictions of negative self-reference in Study 1. **(A)** Illustrates the average choice frequencies of negative self-reference for individuals from the upper (red) and lower quartiles (blue) of the learning rate of negative self-reference (i.e., *α_neg_*). **(B)** Shows the average outcome predictions of negative self-reference (*Q_neg_*) for the same individuals (standard errors are shown in the gray field).

Model-based analyses showed that severity of depressive symptoms is negatively correlated with the learning rate of negative (i.e., *α_neg_*) but not of positive (i.e., *α_pos_*) self-reference (**Figure 3**). In order to examine the influences of the learning rate on the choice behavior and the outcome prediction across trials, we plotted the choice frequency and the mean outcome prediction of negative self-reference (i.e., *Q_neg_*) for individuals who had smaller and greater values (i.e., upper and lower quartiles) of the *α_neg_* parameter (**Figure 4**). Individuals with lower learning rates of negative self-reference showed a delayed shift from negative to positive self-reference after the stimulus-reward contingency reversal (i.e., at the 40th trial). The updating of the outcome prediction was also delayed for the individuals with low learning rates of negative self-reference; the outcome prediction of negative self-reference did not reach zero (not even at the final trial).

In the neutral RL task, all participants except for one^4^ performed better than chance in the acquisition and reversal phases (**Table 1**). We found no significant correlations between the levels of depressive symptoms and the choice frequency of the “B” option (|*r*| s < 0.22, for the acquisition and reversal phases) and the model-based learning parameters (|*r*| s < 0.11, for *α_B_, α_A_*, and *β*). These results suggest that individual differences in depressive symptoms do not significantly affect performance in the neutral RL task.

Next, we performed a regression analysis, in which depressive symptoms were predicted by all the six learning parameters from the emotional and neutral RL tasks. Some of the learning parameters were moderately correlated with each other (*r* = 0.58, for *α_neg_* and *α_pos_*; *r* = 0.52, for *α_B_* and *α_A_*); therefore, the unique association between depressive symptoms and the learning rate of negative self-reference needs to be tested after controlling for the inter-parameter correlations. The results (**Table 3**, Model 1) revealed that the learning rate of negative self-reference remained a significant predictor, whereas other learning parameters did not have significant effects on depressive symptoms. These results suggest that the difficulty in updating outcome predictions is more outspoken for negative self-reference than for positive and neutral stimuli.

**Table 3:** Multiple regressions predicting the severity of depressive symptoms (log-transformed) by learning parameters of the emotional and neutral reversal learning (RL) tasks (Model 1) and rating scores of self-attributes (Model 2) in Study 1.

	Model 1			Model 2		
	B	95% CI	*p*	B	95% CI	*p*
Emotional RL task						
Learning rate—negative (*α_neg_*)	-1.03	[-1.97, -0.08]	0.03	-0.58	[-1.37, 0.20]	0.14
Learning rate—positive (*α_pos_*)	0.45	[-0.39, 1.30]	0.28	0.14	[-0.55, 0.84]	0.68
Exploitation (*β*)	-0.01	[-0.03, 0.01]	0.43	0.00	[-0.02, 0.02]	0.91
Neutral RL task						
Learning rate—B (*α_B_*)	-0.36	[-1.21, 0.50]	0.41	-0.25	[-0.95, 0.46]	0.48
Learning rate—A (*α_A_*)	0.06	[-0.79, 0.91]	0.88	0.22	[-0.47, 0.91]	0.52
Exploitation (*β*)	0.01	[-0.01, 0.03]	0.27	0.00	[-0.01, 0.02]	0.78
Applicability ratings						
Negative self-attributes	–	–	–	0.67	[0.29, 1.05]	<0.01
Positive self-attributes	–	–	–	-0.05	[-0.43, 0.33]	0.80
*R^2^*	0.22			0.53		

Finally, we examined the mediational role of the applicability of self-attributes in the relationship between the low learning rate of negative self-reference and depressive symptoms. Because the low learning rate of negative self-reference increases the chance to be confronted with negative aspects of self, it would reinforce one’s negative self-view and be further associated with depressive symptoms. To test this possibility, we first calculated correlations between the learning rate of negative self-reference and the average rating scores (applicability) of negative and positive self-attributes. The learning rate of negative self-reference had a marginally significant correlation with the applicability of negative (*r* = -0.27, *p* = 0.09) but not positive self-attributes (*r* = 0.08, *p* = 0.64). Second, we estimated a regression model similar to Model 1, in which the applicability of negative and positive self-attributes were added to predict depressive symptoms. The results (**Table 3**, Model 2) showed that the applicability of negative self-attributes was the only significant predictor, which deprived the explanatory power of the learning rate of negative self-reference. The indirect effect (Baron and Kenny, 1986; Preacher and Hayes, 2008), which was calculated by multiplying (a) the effect of the learning rate of negative self-reference on the applicability of negative self-attributes and (b) the effect of the applicability of negative self-attributes on depressive symptoms, was -0.385 (*p* = 0.097; 95% CI [-0.972, 0.079] estimated by bootstrapping of 1000-time resampling). Although the indirect effect was only marginally significant, this result suggests that self-verification can at least in part explain the delayed update of the prediction outcome of negative self-reference in individuals with depressive symptoms.

### Discussion

Study 1 examined the individual differences of depressive symptoms in reward-guided learning of emotional self-reference. In the baseline task, individuals with higher levels of depressive symptoms showed a greater preference for negative self-reference, which replicates the findings of previous studies that suggested excessive negativity bias and a lack of positivity bias in depression (e.g., Mezulis et al., 2004). Regardless of these differences in the baseline preference, all individuals successfully learned the association between positive self-reference and reward after the reversal of the stimulus-reward contingencies in terms of the choice frequency of negative self-reference. However, as we hypothesized, individuals with higher levels of depressive symptoms had lower learning rates of negative self-reference, implying that those individuals have difficulty adjusting their outcome predictions of negative self-reference to the volatility of the action-outcome contingencies.

We also found a correlation (although only marginally significant) between the learning rate of negative self-reference and the applicability of negative self-attributes. Furthermore, the applicability of negative self-attributes had a mediating role (again marginally significant) in the association between impaired updating and depressive symptoms; that is, people with difficulty in updating the reward prediction of negative self-reference also tended to have negative self-views and, at the same time, tended to suffer from increased depressive symptoms. This mediation could be interpreted as indicating that a low learning rate increases the chance to be exposed to negative self-affirmative information. This could, on its turn, reinforce negative self-views and lead to depressive symptoms (e.g., Evraire and Dozois, 2011). However, it should be noted that the small sample size of Study 1 may have limited the power to detect the statistical significance for this mediation.

One important limitation of Study 1 was that the stimulus-reward contingencies were changed only once across the trials. Therefore, the emotional RL task could not fully capture the process in which participants, particularly those with higher levels of depressive symptoms, increase the reward expectancy of negative self-reference. Since individuals with higher levels of depressive symptoms had greater preference for negative self-reference in the baseline task, they mostly chose negative self-reference in the first trial of the emotional RL task. These individuals did not need to newly learn and establish the association between negative self-reference and reward in the acquisition phase. Thus, it is possible that the learning parameters estimated in Study 1 might not reflect the process of learning the association between negative self-reference and reward.

## Study 2

In order to overcome the just mentioned limitation of Study 1, we modified the emotional RL task by adding a second reversal phase, wherein negative self-reference is more associated with reward than with punishment, and positive self-reference is more associated with punishment than with reward (**Figure 2B**). In this setting, all participants had to learn (a) the association between positive self-reference and reward in the first reversal phase, and (b) the association between negative self-reference and reward in the second reversal phase. The initial choice in each reversal phase is determined by the learned contingencies in the previous phase (e.g., the first choice in the second reversal phase should be “positive,” which was reinforced in the first reversal phase); therefore, we could examine the process of shifting from positive to negative self-reference independent of the preference that the participants originally exhibited. In line with the results of Study 1, we predicted that individuals with higher levels of depressive symptoms would have lower learning rates (i.e., slower value update) for negative self-reference.

### Method

#### Participants and Procedures

Forty-four participants (23 men and 21 women; mean age = 19.4 years, *SD* = 1.2 years) were recruited from a large sample pool of undergraduate students from the University of Tokyo. The procedure of Experiment 2 was identical to that of Experiment 1 except for the reinforcement schedule in the RL tasks (**Figure 2**): the first one-third of the trials (26 trials) were the acquisition phase, in which the “negative” option was associated with 80% of reward and 20% of punishment; the second one-third of the trials were the first reversal phase, in which the “negative” option was associated with 20% of reward and 80% of punishment; and the last one-third of the trials were the second reversal phase, in which the “negative” option was again associated with 80:20% of reward and punishment. Therefore, the stimulus-reward contingencies were reversed twice across the 78 trials. All participants completed the baseline task without reward/punishment feedback (20 trials), following which they completed the emotional and neutral RL tasks in a counterbalanced order. It is to be noted that the learning parameters (learning rates and exploitation parameter) were not influenced by the task order, *t*s < 1.50, *p*s > 0.14. The mean CES-D score was 12.8 (*SD* = 8.5) and the Cronbach’s alpha was 0.86. Fourteen participants showed a level of symptoms above the clinical cutoff (>15) of the CES-D.

### Results and Discussion

In the baseline task, individuals with higher levels of depressive symptoms chose negative self-reference more frequently (*r* = 0.44, *p* < 0.01), which indicates that those individuals had greater preference for negative self-reference before engaging in the learning tasks. Furthermore, they rated the negative attributes to be more applicable (*r*s = 0.70 and 0.69, *p* < 0.01, in the baseline and emotional RL tasks, respectively) and the positive attributes to be less applicable to themselves (*r*s = -0.51 and -0.59, *p* < 0.01 in the baseline and emotional RL tasks, respectively).

In the emotional RL task, all except for three participants^5^ performed better than chance in the acquisition, first reversal, and second reversal phases (**Table 2**). Similar to Study 1, we examined the frequency of the “*not at all*” responses in the self-applicability rating, which was only 9.5% in Study 2. This result suggests that in most trials, participants endorsed the aspects of the self that correspond to the presented rating stimuli.

**FIGURE 5:**
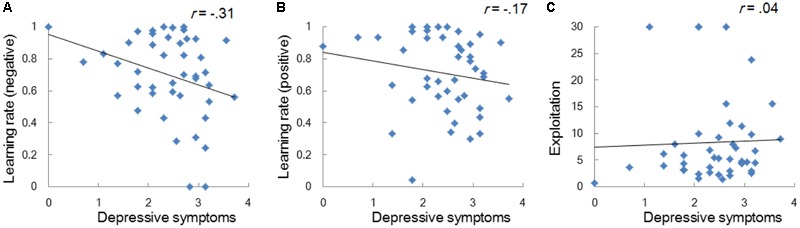
Correlations between depressive symptoms and the learning rate of negative **(A)** and positive self-reference **(B)**, and the exploitation parameter **(C)** in Study 2. The depressive symptoms score (the CES-D score) was log transformed.

**FIGURE 6:**
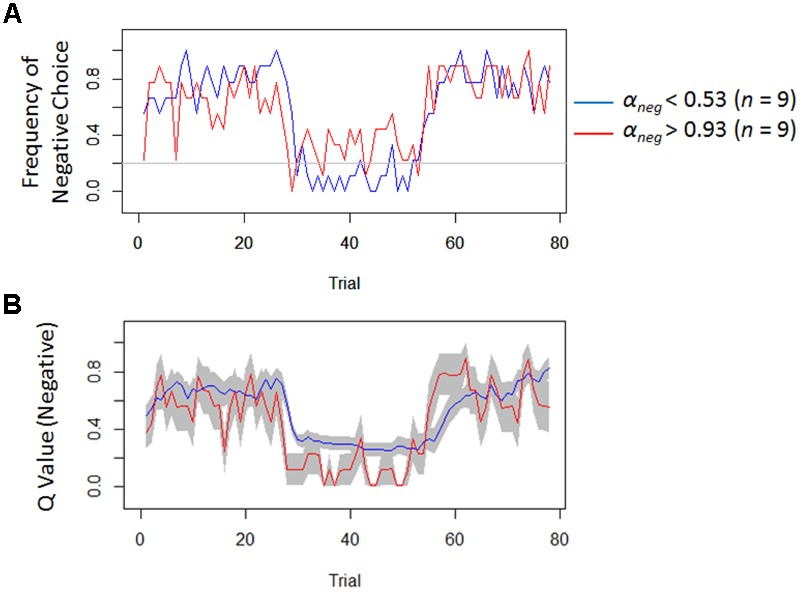
Frequency of negative choice and average outcome predictions of negative self-reference in Study 2. **(A)** Presents the average choice frequencies of negative self-reference for individuals from the upper (red) and lower quartiles (blue) of the learning rate of negative self-reference (i.e., *α_neg_*). **(B)** Shows the outcome predictions of negative self-reference (*Q_neg_*) for the same individuals (standard errors are shown in the gray field).

Regardless of the differences in the choice frequency between Experiments 1 and 2, the model-based analyses replicated the associations between depressive symptoms and learning parameters. We used the same Q-learning model as in Study 1, in order to estimate the learning rates and exploitation parameters (i.e., *α_neg_, α_pos_*, and *β*). As we hypothesized, the learning rate of negative self-reference was the only parameter that was significantly correlated with depressive symptoms (**Figure 5**). To visualize the choice behaviors and outcome predictions across trials, we plotted the average choice frequency of negative self-reference and mean *Q_neg_* values for individuals with lower and higher learning rates of negative self-reference (**Figure 6**). The lower learning rate of negative self-reference was associated with slower update of the outcome predictions of negative self-reference^6^.

In the neutral RL task, most of the participants performed better than chance in all the three learning phases, although six participants failed to exceed a chance level either in the acquisition or in the first reversal phase. Similar to the results of Experiment 1, none of the choice frequency (|*r*| s < 0.24, *p*s > 0.12) and learning parameters (|*r*| s < 0.08, *p*s > 0.60) were significantly correlated with depressive symptoms. These null correlations suggest that individual differences in depressive symptoms did not significantly affect the learning process for the neutral stimuli.

We also performed a regression analysis predicting depressive symptoms by the six learning parameters of the emotional and neutral RL tasks to control the inter-parameter correlations (**Table 4**, Model 1). The results showed that the learning rate of negative self-reference was the only significant predictor of depressive symptoms. These results replicate and extend the findings from Study 1, suggesting that depressive symptoms are associated with delayed update of outcome predictions of negative self-reference, even when the task included a second reversal phase that required shifting from positive to negative self-reference.

**Table 4:** Multiple regressions predicting the severity of depressive symptoms (log-transformed) by learning parameters of the emotional and neutral reversal learning (RL) tasks (Model 1) and rating scores of self-attributes (Model 2) in Study 2.

	Model 1			Model 2		
	B	95% CI	*p*	B	95% CI	*p*
Emotional RL task						
Learning rate—negative (*α_neg_*)	-1.43	[-2.78, -0.09]	0.04	-0.44	[-1.46, 0.58]	0.39
Learning rate—positive (*α_pos_*)	0.41	[-0.97, 1.79]	0.55	0.30	[-0.70, 1.3]	0.55
Exploitation (*β*)	0.00	[-0.02, 0.03]	0.74	0.01	[-0.02, 0.03]	0.62
Neutral RL task						
Learning rate—B (*α_B_*)	-0.25	[-1.15, 0.65]	0.58	-0.48	[-1.13, 0.17]	0.14
Learning rate—A (*α_A_*)	0.99	[-0.36, 2.34]	0.14	0.70	[-0.27, 1.67]	0.15
Exploitation (*β*)	-0.01	[-0.03, 0.02]	0.63	0.00	[-0.02, 0.01]	0.55
Ratings						
Negative self-attributes	–	–	–	0.70	[0.34, 1.06]	<0.01
Positive self-attributes	–	–	–	-0.40	[-0.75, -0.06]	0.02
*R^2^*	0.15			0.59		

Finally, we tested the mediational effects of the applicability of negative and positive self-attributes on the relationship between the low learning rate of negative self-reference and depressive symptoms. We found significant correlations between the learning rate of negative self-reference and the average rating scores (applicability) of the negative (*r* = -0.35, *p* = 0.02) and positive self-attributes (*r* = 0.30, *p* = 0.04). An additional regression analysis revealed that the applicability of the negative and positive self-attributes were significant predictors of depressive symptoms, which reduced the explanatory power of the learning rate of negative self-reference to a non-significant level (**Table 4**, Model 2). The indirect effect of the learning rate of negative self-reference on depressive symptoms was -0.527 (*p* = 0.035; 95%CI [-1.493, -0.152], estimated by bootstrapping of 1000-time resampling) mediated by negative self-attributes, and -0.267 (*p* = 0.109; 95%CI [-0.860, -0.016]) mediated by positive self-attributes^7^. These results suggest the mediational role of a negative self-image in the association between the low learning rate and depressive symptoms; that is, people with updating difficulties seem to be faced with negative self-affirmative information, which may lead to negative self-image and depressive symptoms.

## General Discussion

The present research provides empirical evidence for the learning hypothesis of the self-negativity bias in depression, stating that individuals with depressive symptoms have difficulty in adjusting their outcome predictions of negative self-reference to the volatility of the environment. The two studies consistently showed a significant correlation between depressive symptoms and a low learning rate of negative self-reference, which represents a significant delay in updating the outcome predictions of negative self-reference after the reversal of stimulus-reward contingencies. Our findings shed new light on the possible learning mechanism underlying the self-negativity bias in individuals with depressive symptoms. Existing theories and research have exclusively focused on dysfunctions in attention and (working) memory, suggesting that depressive cognitions can be characterized by impaired attentional disengagement from and impoverished inhibitory control of negative self-referent information processing (e.g., Gotlib and Joormann, 2010). This cognitive account of depression provides a good theoretical basis, explaining why it is difficult for depressed people to stop negative thinking once it has started. However, until now, it was still unclear why those individuals voluntarily engaged in and often preferred negative to positive self-reference even when it brings harmful consequences (Coyne, 1976; Swann et al., 1992; Giesler et al., 1996). In the present studies, we showed that individuals with depressive symptoms are inflexible in updating and adjusting their outcome predictions of negative self-reference in a volatile environment in which the outcome of negative self-reference is variable over time. This result implies that those individuals tend to keep a high reward-expectancy for negative self-reference even after the reversal of the actual action-outcome contingencies, which support the persistent belief that negative self-reference is more rewarding (and more preferred) than positive self-reference.

The difficulty in updating outcome predictions of negative self-reference was associated with the extent to which one possesses negative self-images. Both Studies 1 and 2 showed (marginally) significant negative correlations between the learning rate of negative self-reference and the applicability of negative self-attributes. Follow-up mediation analyses (particularly in Study 2) indicated an indirect effect of the low learning rate on depressive symptoms via negative self-attributes. The low learning rate of negative self-reference increases the chance to be faced with negative aspects of self, which may result in depressive symptoms. However, it could also be the other way around. More precisely, people with depressive symptoms may have a higher motivation to persist in approaching negative self-referent information and therefore refrain from updating on the basis of reward predictions. The self-verification theory of depression indeed holds that confirmation of one’s self-image promotes a sense of self-coherence and fosters the perception that one is true to oneself (Swann et al., 1992; Giesler et al., 1996). In line with these arguments, a recent study showed that individuals with higher levels of depressive symptoms have greater preference toward negative self-reference; conversely, those with lower levels of depressive symptoms tend to avoid negative self-reference even though they lose an opportunity to obtain a monetary reward by doing so (Takano et al., 2016). Facing non-self-affirmative information would be a relatively aversive experience that arouses a sense of self-discrepancy and triggers an avoidance reaction, for example, when individuals with depressive symptoms have to rate positive attributes (e.g., “Happy”) as being “not applicable to me.” Thus, it is possible that rating self-attributes *per se* would be an extra reward and/or extra punishment, by confirming negative and disconfirming positive self-images for individuals with depressive symptoms (cf. Watson et al., 2008). It is, however, important to note that we relied on a cross-sectional design in both studies. We therefore cannot specify the causal directions of the detected mediation^8^. Also note that some effects included in the mediation were only marginally significant (e.g., the indirect effect of the learning rate in Study 1). Replication and extension are thus needed with more rigorous (e.g., longitudinal) designs to establish the mediational association.

The avoidance mechanism is worth mentioning here for another reason. In our task, participants could avoid positive self-reference by choosing negative self-reference. In this respect, it is of special interest that the Q-learning model assumes that the outcome prediction of an unchosen option is not updated, which means that avoiding positive self-reference maintains low levels of reward expectancy of positive self-reference (however, see e.g., Schlagenhauf et al., 2014, for variants of the Q-learning model). This might be a good analogy to the avoidance mechanisms at play in depression (e.g., Jacobson et al., 2001; Ramnerö et al., 2015): inactivity and avoidance in depression are argued to reduce the opportunity to experience circumstances that would lead to environmental reward and reinforcement^5^.

It is worth noting that, across the two studies, we found no significant association between depressive symptoms and the learning rate for positive self-reference^9^. This null association suggests that the reward-guided learning of positive self-reference may not be disturbed in individuals with depressive symptoms, and thus, that those individuals can come to choose positive instead of negative self-reference given a high enough number of reinforced trials. This valence-specific effect should be interpreted with caution though. First, one might argue that a single (but not double) learning rate would be sufficient to describe the participants choice behavior (see Model Comparison) and that the current results can be attributed to a general deficit in the updating function that is not specific for negative self-reference. However, the following arguments go against this criticism: (a) the model with a single learning rate is nested in the model with two learning rates, so if the single learning rate would have provided a more appropriate fit for a given participant, the estimates for the two learning rates should have been more or less equal as in the two-learning-rate model; (b) if a general deficit is at play, both the learning rates for the negative and positive response options should have been correlated with depressive symptoms. Second, one may argue that the lack of a control condition in which the positive response option is initially reinforced could be considered to give room to the alternative interpretation that depressive symptoms are associated with a difficulty in updating the reward predictions for the initially learned response, but not for negative self-reference *per se*. However, our data of the neutral task with the non-emotional and non-self-referent stimuli suggests that this is not the case. Still, it would be important to examine this alternative interpretation more directly by including this control condition in follow-up research.

Making abstraction of these alternative interpretations, our findings on the valence specificity could have important implications for recent cognitive-bias-modification (CBM) approaches, which aim to alleviate depressive symptoms by correcting negative attentional and interpretational biases (e.g., Hertel and Mathews, 2011). Although the efficacy of CBM interventions is still controversial (Hakamata et al., 2010; Hallion and Ruscio, 2011; Beard et al., 2012; Cristea et al., 2015), our data highlight the potential of reward-guided reinforcement learning as a novel method to correct self-negativity bias and to enhance self-positivity bias in depression (cf. Hertel and Mathews, 2011; Lau, 2013). Future research could focus on how to consolidate the learned association between positive self-reference and reward, because that association appeared relatively fragile in the present study (cf. the second reversal phase in Study 2).

It is tempting to consider the current results as an indication that negative self-referent thinking is a mental habit in depression, as discussed in the literature on depressive rumination (Hertel, 2004; Verplanken et al., 2007; Watkins and Nolen-Hoeksema, 2014; Ramnerö et al., 2015). Indeed, our results showed that participants with high levels of depressive symptoms continue to select negative self-reference even after the contingency reversal. This result appears to be consistent with the hypothesized “habitual” nature of depressive rumination, which is characterized by the difficulty to oppose depressive rumination despite its negative outcomes (Hertel, 2004). However, we should be careful to interpret the current results as evidence for the habitual nature of rumination because of two reasons. First, on a conceptual level, depressive thinking or rumination is still different from choosing a “negative” option in a decision-making task. Second, to conclude that a behavior is a habit (Heyes and Dickinson, 1990; de Wit and Dickinson, 2009), researchers need to test the behavior under conditions of (a) contingency degradation (i.e., belief criterion) and (b) outcome devaluation (i.e., desire criterion). Although the emotional RL task taps into the belief criterion, it does not examine whether negative self-reference in depression meets the desire criterion, that is, whether participants continue the reinforced response after outcome devaluation by, for example, saturation (Valentin et al., 2007) or instructed devaluation (de Wit et al., 2007).

Even if negative self-reference would be a habit-like behavior that is no longer driven by the goal or desired outcome that initially installed it, it might still be changed by identifying and manipulating “hidden goals.” Moors et al. (in press) have indeed proposed that a single action may have multiple outcomes. In our experiments, one and the same response option (or action) might have had two goals; one obvious goal was to obtain monetary reward, whereas another hidden goal might have been to be consistent with negative or positive self-views. In the first reversal phase, people with depressive symptoms, who tend to have negative self-views, might have experienced a conflict between these two goals, i.e., earning money but being exposed to positive self-images. Our findings could be taken as evidence that the latter hidden goal weakens the reinforcement for the “positive” response for those individuals. Therefore, we can expect that identifying and changing the hidden goal, or manipulating the negative self-views (e.g., Serrano et al., 2004), would be therapeutically beneficial, because engaging in negative self-referent thinking would no longer satisfy this hidden goal of being consistent with negative self-views. Watkins and Nolen-Hoeksema (2014) also proposed an intervention to reduce rumination as a mental habit, which involves the repeated practice of using alternative coping strategies in response to an identified habit-triggering context (e.g., when lying in bed).

It is also important to note that we did not find any significant associations between depressive symptoms and the learning parameters of the neutral RL task. Previous studies using the neutral RL task have suggested aberrant reward-punishment sensitivity in clinical levels of depression (Murphy et al., 2003; Taylor Tavares et al., 2008; Dombrovski et al., 2010; Robinson et al., 2012), implying that patients with depression shift from one to the other option more often than do healthy controls when receiving probabilistic negative feedback (i.e., punishment after a correct response) or when receiving unexpected reward. One critical difference between the previous and current studies is that our sample consisted of non-clinical university students. In our data, around 30% of participants showed a level of depressive symptoms above the clinical cutoff of the CES-D (>15), which is comparable to the general prevalence rate in university students (i.e., 30.6%, Ibrahim et al., 2013). Given the continuity of depression between clinical and non-clinical samples (Flett et al., 1997), we would argue that our results provide a solid basis for linear predictions for more severe levels of depression. However, the absolute number of participants who were at a clinical level of depressive symptoms was relatively small in the current sample (24 participants across two studies). Therefore, future research should confirm this assertion in a sample of clinically depressed people.

Another remaining question (particularly of Study 2) is why depressive symptoms are associated with a delay in re-learning that negative self-reference is rewarded and not only punished. Our results (see **Figure 6B**, the second reversal phase) seem to indicate that it takes a relatively long time for people with depressive symptoms to acquire a preference toward negative self-reference^10^, which might reflect a blunted sensitivity to external reward and punishment in updating belief about negative self-reference. However, it should be noted that the delay that we observed here might be merely due to the model assumption that the learning rate had to be equal between rewarded and punished trials. This constraint was installed because a model with four learning rates (i.e., negative and positive/rewarded and punished trials) had too many free parameters (leading to a convergence issue). Since some studies have suggested that the learning rate can be different between rewarded and punished trials (e.g., Dombrovski et al., 2010), future research needs to dissociate the four different updating processes (i.e., rewarded versus punished and negative versus positive self-reference) to specify which delay best models depression. This could be achieved by estimating a model with learning rates for rewarded and punished trials in an experiment with two between-person conditions: a condition with a negative-to-positive (i.e., learning that the negative is punished and the positive is rewarded after the contingency reversal) and condition with a positive-to-negative transition (i.e., learning that the positive is punished, and the negative is rewarded after the contingency reversal).

## Conclusion

The present research is the first to provide evidence that individuals with depressive symptoms have difficulty updating their outcome predictions of negative self-reference in a volatile environment. This inflexibility in updating outcome predictions could contribute to excessive focus on negative aspects of the self, that is, to self-negativity bias in depression. Furthermore, the difficulty in updating the reward prediction of negative self-reference is correlated with the negative self-image that individuals with depressive symptoms often possess. The consistency between their negative self-images and negative self-reference (and discrepancy between their negative self-image and positive self-reference) may be associated with the delayed shift from negative to positive self-reference. We believe that the reinforcement learning and model-based approach could be a promising starting point to reveal the mechanisms of the persistence and repetitiveness of depressive cognitions.

## Author Contributions

YI and KT contributed to the conception and designed the studies; YI collected the data; KT, YB, FR, and YT analyzed and/or interpreted the data; KT drafted the work, and YI, YB, FR, and YT revised it for important intellectual contents; All authors approved the final version of the manuscript, and agreed to be accountable for all aspects of the work and ensure that any questions related to the accuracy.

## Conflict of Interest Statement

The authors declare that the research was conducted in the absence of any commercial or financial relationships that could be construed as a potential conflict of interest.

## Footnotes

1 These attributes were presented in the form of a phrase or sentence. Because of the language differences, the examples are shown as if they were a single-word stimuli; however, for example, the stimulus “happy,” was more like “(I) am happy” in the original (Japanese) form. The full list of the stimuli is available on request from the corresponding author.

2 Providing reward/punishment feedback according to the applicability rating would make the rating scores less reliable. If reward was provided only for an “applicable” response, participants would be motivated to make the “applicable” response even when the presented attribute is not applicable to them.

3 The data from five participants who always chose either the “negative” or “positive” option over the trials were not used in calculating these correlations.

4 This participant had 82.5 and 52.5% of “B” choices in the acquisition and reversal phases, respectively. However, results remained unchanged after eliminating the data pertaining to this participant.

5 Results remained unchanged after excluding the data of these participants. Similarly, in the neutral RL task, six participants failed to exceed a chance level. These data did not influence the results.

6 The low *α_neg_* value was also associated with delayed update of the outcome predictions of negative self-reference in the second reversal phase. However, this delay did not appear to influence the choice behavior itself (**Figure 5A**), because the re-learning process after the second contingency reversal is mainly controlled by the learning rate of positive, instead of negative self-reference. In several initial trials of the second reversal phase, participants mostly continued to select the positive option as it had been associated with reward in the first reversal phase. Thus, the outcome prediction is more frequently updated for the positive than negative option under the current Q-learning model assumption (the outcome prediction is updated only for the chosen option); in other words, the choice behaviors in the second reversal phase are dominantly influenced by the increase in reward prediction for the positive option. Our results showed that the learning rate of the positive option is not associated with depressive symptoms; therefore, the process of re-learning the association between positive self-reference and reward should not be altered even in individuals with high levels of depressive symptoms.

7 These mediation effects are estimated in a single model with multiple mediators (i.e., the negative and positive self-applicability ratings).

8 We also tested a “feedback” model with the reversed path direction, i.e., depressive symptoms -> negative self-attributes -> the learning rate. Here, the indirect effect was -0.01 (*p* = 0.88, 95%CI [-0.14, 0.11]) in Study 1 and -0.05 (*p* = 0.26, 95%CI [-0.16, 0.04]) in Study 2. These null effects suggest that our hypothesized mediational association (i.e., the learning rate -> negative self-attributes -> depressive symptoms) fits the data better than the alternative mediation with the opposite direction.

9 As additional evidence, we found that depressive symptoms are significantly correlated with a difference score between the negative and positive learning rates in the pooled data of Studies 1 and 2 (*r* = -0.24, *p* = 0.027). This significant correlation suggests that depressive symptoms are associated with a large delay in updating outcome predictions for negative relative to positive responses.

10 Note that in the acquisition phase (before any contingency reversal), people with higher levels of depressive symptoms had already shown greater preference toward the negative option (see also the results of the baseline tasks). These individuals did not need to newly “learn” the association between negative self-reference and reward in the acquisition phase.
